# Expression of the phosphodiesterase BifA facilitating swimming motility is partly controlled by FliA in *Pseudomonas putida* KT2440

**DOI:** 10.1002/mbo3.402

**Published:** 2016-09-23

**Authors:** Yujie Xiao, Huizhong Liu, Hailing Nie, Shan Xie, Xuesong Luo, Wenli Chen, Qiaoyun Huang

**Affiliations:** ^1^ State Key Laboratory of Agricultural MicrobiologyHuazhong Agricultural UniversityWuhanChina; ^2^ Key Laboratory of Arable Land Conservation (Middle and Lower Reaches of Yangtze River)Ministry of AgricultureCollege of Resources and EnvironmentHuazhong Agricultural UniversityWuhanChina

**Keywords:** BifA, C‐di‐GMP, feedback regulation, FliA, swimming motility

## Abstract

Flagella‐mediated motility is an important capability of many bacteria to survive in nutrient‐depleted and harsh environments. Decreasing the intracellular cyclic di‐GMP (c‐di‐GMP) level by overexpression of phosphodiesterase BifA promotes flagellar‐mediated motility and induces planktonic lifestyle in *Pseudomonas*. The mechanism that regulates expression of *bifA* gene was poorly studied. Here we showed that expression of BifA was partly controlled by flagellar sigma factor FliA (σ^28^) in *Pseudomonas putida* KT2440. FliA deletion led to an approximately twofold decrease in transcription of *bifA*. 5′ race assay revealed two transcription start points in *bifA* promoter region, with the putative σ^70^ and σ^28^ promoter sequences upstream, respectively. Point mutation in σ^28^ promoter region reduced transcriptional activity of the promoter in wild‐type KT2440, but showed no influence on that in *fliA* deletion mutant. FliA overexpression decreased the intracellular c‐di‐GMP level in a BifA‐dependent way, suggesting that FliA was able to modulate the intracellular c‐di‐GMP level and BifA function was required for the modulation. Besides, FliA overexpression enhanced swimming ability of wild‐type strain, while made no difference to the *bifA* mutant. Our results suggest that FliA acts as a negative regulator to modulate the c‐di‐GMP level via controlling transcription of *bifA* to facilitate swimming motility.

## 1 Introduction

Flagella‐mediated motility is an important capability of many bacteria to explore the environment for nutrients and escape from hostile environmental conditions. In most flagellated bacteria, more than 50 genes are typically involved in flagella production, a complex process that requires coordination in space and time (Liu & Ochman, 2007). In the genus *Pseudomonas*, the master regulator FleQ, together with RpoN (σ^54^), induces expression of the class II flagellar genes including *fleSR*, which encode a two‐component system that in turn activates class III genes in concert with RpoN. The sigma factor FliA is required for transcription of class IV flagellar genes including *fliC*, which encodes the major flagellin subunit (Dasgupta et al., 2003; Liu & Ochman, 2007). Although the flagellar machinery endows cells with a phenomenal survival device, it also consumes much of the metabolic currency to synthesize and fuel such a vigorous nanomotor (Martínez‐García, Nikel, Chavarría, & de Lorenzo, 2014). Several feedback regulations in flagella synthesis and rotation have been reported and believed to function as insurances to avoid costly production of unnecessary flagellar subunits or flagellar rotation under unnecessary conditions (Aldridge & Hughes, 2002). For example, the interaction between FliA and the cytoplasmic anti‐sigma factor FlgM depresses transcription of class IV flagellar genes by inhibiting the FliA‐RNA polymerase association until completion of the hook‐basal body complex, at which point the anti‐sigma factor is secreted (Chadsey, Karlinsey, & Hughes, 1998; Frisk, Jyot, Arora, & Ramphal, 2002). Besides, the c‐di‐GMP‐binding protein, YcgR interacted with the flagellar switch‐complex proteins FliG and FliM, caused reduced flagellar rotation and swimming ability, most strongly in the presence of c‐di‐GMP in enteric bacteria (Fang & Gomelsky, 2010; Paul, Nieto, Carlquist, Blair, & Harshey, 2010).

In diverse bacterial species, the secondary message c‐di‐GMP contributes to regulate bacterial swimming motility (Hengge, 2009; Jenal & Malone, 2006). Increasing the concentration of intracellular c‐di‐GMP by overexpression of diguanylate cyclase (DGC) enzymes promotes expression of genes for biofilm components and leads to biofilm formation, while low levels caused by phosphodiesterase (PDE) activity induces a planktonic lifestyle with highly motile cells. It has been reported that c‐di‐GMP modulates the transition between planktonic and biofilm life styles through the transcription factor FleQ in *Pseudomonas* species (Baraquet & Harwood, 2015; Hickman & Harwood, 2008; Martínez‐Gil, Ramos‐González, & Espinosa‐Urgel, 2014; Xiao et al., 2016). *BifA* encoding a genus‐specific PDE, which had been identified in *Pseudomonas fluorescens* (Martínez‐Granero et al., 2014), *Pseudomonas aeruginosa* (Kuchma et al., 2007), and *P. putida* (Jiménez‐Fernández, López‐Sánchez, Calero, & Govantes, 2015), were found to regulate the intracellular c‐di‐GMP pool. BifA overexpression caused increased swimming motility and decreased biofilm formation. This suggested that this gene play a central role in regulating bacterial motility via the c‐di‐GMP pathway. Therefore, figure out the regulation of *bifA* will deepen our understanding on the regulating of *P. putida* KT2440 motility.

Among the known sigma factors, the flagellar regulator FliA control numerous genes other than those involved in flagellar biogenesis in several bacterial species (Claret et al., 2007; Byoung‐Mo Koo, Rhodius, Campbell, & Gross, 2009; Yu, Kibler, & Tan, 2006). For example, in *Escherichia coli* strain LF82, accumulation of FliA leads to a concomitant strong induction of *yhjH*, which encodes a PDE. This implied that FliA putatively functions as a c‐di‐GMP regulator. It is interesting to see that in a previous microarray analysis showed that the transcription of *bifA* in a *P. putida fliA* mutant decreased approximately twofold (Rodríguez‐Herva et al., 2010). Therefore, we hypothesized that the expression of BifA was putatively directly regulated by FilA and FliA possessed an ability to modulate the intracellular c‐di‐GMP level via regulating BifA expression in *P. putida*.

In this study, a set of experiments was carried out to test the hypothesis. Based on the experimental results, we confirm that expression of the BifA is partly controlled by the flagellar sigma factor FliA in *P. putida* KT2440, and FliA overexpression decreases intracellular c‐di‐GMP level in a BifA‐dependent way. By enhancing expression of BifA, FliA acted as a negative regulator to modulate the c‐di‐GMP level to facilitate swimming motility. Our results imply a positive feedback function of FliA in swimming ability regulation.

## 2 Experimental Procedures

### 2.1 Bacterial strains, plasmids, media, and culture conditions

All bacterial strains, plasmids, and primers used in this study are listed in Table 1. The *P. putida* KT2440 and *E. coli* DH5α, S17‐1/λ*pir* strains were routinely cultured at 28°C and 37°C, respectively, on Luria‐Bertani (LB) broth medium, which was solidified with 1.5% agar when necessary. Gentamicin was used at 40 μg/ml for *P. putida* and at 20 μg/ml for *E. coli*. Kanamycin was used at 50 μg/ml and chloramphenicol at 25 μg/ml. For expression plasmids harboring the *ptac* promoter, isopropyl β‐D‐thiogalactoside (IPTG) was added to cultures at a 0.4 mM final concentration when needed. Gentamicin and kanamycin were used for plasmid maintenance in *P. putida*.

**Table 1:** Bacterial strains, plasmids and primers used in this work

Strains/plasmids/primers	Description	Source or reference
*Escherichia coli* strains		
DH5α	λ‐Φ80dlacZΔM15Δ(lacZYA‐argF)U196recA1endA1 hsdR17(rK‐ mK ‐) supE44 thi‐1 gyrA relA1	Invitrogen Corp
S17‐1/λpir	RK2 tra regulon, pir, host for pir‐dependent plasmids	(Simon, Priefer, & Pühler, 1983)
*Pseudomonas putida* strains		
KT2440	Wild type	(Bagdasarian et al., 1981)
Δ*fliA*	Unmarked *fliA* deletion mutant	This work
Δ*bifA*	Unmarked *bifA* deletion mutant	This work
Plasmids		
pVLT33	Broad‐host‐range cloning vector, containing *pTac* promoter, Km^r^	(De Lorenzo, Eltis, Kessler, & Timmis, 1993)
pVLT33‐*fliA*	Complete *fliA* gene in pVLT33, Km^r^	This work
pBBR1‐401	Knockout vector, derived from pBBR1‐MCS5, with origin fragment replaced by ori R6K origin fragment	(Xiao et al., 2016)
pBBR401‐fliAUP‐DW	Suicide plasmid containing up and down homologous region of *fliA*	This work
pBBR401‐bifAUP‐DW	Suicide plasmid containing up and down homologous region of *bifA*	This work
pBBR–*lacZ*	Derived from pBBR1‐MCS5, harbors a promoterless *lacZ* gene, Gm^r^	(Xiao et al., 2016)
pBBR‐*bifA*pro‐*lacZ*	Reporter plasmid constructed by ligating *bifA* promoter DNA to the promoterless *lacZ* gene in pBBR–*lacZ*, Gm^r^	This work
Primers		
fliAups	CCGCTCGAGGCTGTGCGTCAGGAAACTG	
fliAupa	CCGGAATTCTCGGTGAGGTGCTGGGT	
fliAdowns	CCGGAATTCGCGTAGCGTTCGATCAGC	
fliAdowna	CGCGGATCCGACGAGCCCAGCGAAGAG	
fliAS	CGCGGATCCCCTCCAAGCAGTCTCAAACG	
fliAA	CCGGAATTCATGAACGCCAGCGGCTTC	
bifApros	AAAACTGCAGGACCCTGAGCAACTTGACC	
bifApros	CGGGGTACCGCGGCATCTTCAACAACG	
bifAups	CTAGTCTAGAGTTGACAGGCTCGGCTTCC	
bifAupa	CGCGGATCCCGGGTTTCGGTGTCCTGA	
bifAdowns	CGCGGATCCGCATGGCATCATTTTGCC	
bifAdowna	CCGCTCGAGCCCTGCGGAACCTCTATT	
qpcrbifAs	CGCTTGTTTGAGGAAGTG	
qpcrbifAa	GCAGGAAACCTACATCGT	
‐35bifAs	GCGGATTGCGGGGTCAGGCAAC	
‐35bifAa	ATGCGCTGTATCAATCTTCC	
‐10bifAs	GTTGCACACTGCTAATGGTCGG	
middlebifAs	AACCGATACACTGCTA	
middlebifAa	CGCAACTTGAATGCGCTG	
‐10bifAs	TTGCCTGACCCCGCAAC	
bifA‐race	TGATTTGCGCACAGCTGA	
UP	CTAATACGACTCTATGGGCAAGCAGTGGTATCAACGCAGAGT	

The complementary regions of the primers are indicated by underline.

### 2.2 Plasmid and strain construction

To construct a markerless *P. putida fliA* deletion mutant, ~1,000 bp from the chromosomal regions flanking *fliA* were PCR‐amplified with oligonucleotide pairs fliAups and fliAupa (upstream region), or fliAdowns and fliAdowna (downstream region). The PCR products were ligated into pBBR401 (Xiao et al., 2016), yielding pBBR401‐fliAUP‐DW. Then the final plasmid was transferred to *P. putida* KT2440 by electroporation. Selection of integration gentamicin resistance strain was performed on gentamicin and chloramphenicol double antibiotics plates. After subculture the integration strain in LB medium without antibiotic for ten generations, single colonies were obtained by plate streaking. Then the identity of delete mutants were confirmed by PCR and sequencing. Markerless *bifA* mutant was constructed using the same method.

A DNA fragment containing the complete *fliA* coding region was PCR amplified using oligonucleotides *fliA*S and *fliA*A as primers. The product was digested with EcoRI and BamHI and ligated to pVLT33 to yield pVLT3‐*fliA*. Complementation was accomplished by introducing pVLT33‐*fliA* into *fliA* mutant. To generate the *lacZ* reporter plasmid, about 500‐bp fragment containing the promoter region of relevant gene was obtained by PCR. The PCR product was cloned into vector pBBR‐*lacZ*, which harbors a promoterless *lacZ* gene.

Mutations in the promoter region were generated by inverting PCR with oligonucleotides containing the mutation (‐35bifAs and ‐35bifAa, ‐10bifAs, and ‐10bifAa, middlebifAs and middlebifAa), and pBBR‐*bifA*pro‐*lacZ* was used as template. The fragment was then circled by adding DNA ligase and transformed into *E. coli* S17‐1. Plasmid was transferred to *E. coli* or *P. putida* strains by transformation, biparental mating, or electroporation. All cloning steps involving PCR were verified by sequencing (Tsingke).

### 2.3 Swimming motility assay

Swimming assays were carried out as reported previously (Kuchma et al., 2007). Swimming plates (0.3% agar) consisted of M63 medium supplemented with glucose, MgSO_4_, and CAA were toothpick‐inoculated with fresh colonies and incubated for 20 h at 28°C. Then digital photographs were taken, and the diameters of the swim zones were measured in millimeters and averaged over four replicate platings.

### 2.4 Biofilm formation analysis

The quantitative analysis of biofilm forming was performed using crystal violet (CV) staining as previously described (O'Toole & Kolter, 1998). The total of 1% (v/v) of the bacterial culture (OD_600_ ≈ 1.0) was inoculated into 50 ml LB medium, after 8 h growth at 28°C with 180 rpm shaking, 0.4 mM (final concentration) IPTG was added to the culture. Then the bacterial culture (OD_600_ ≈ 1.5) was aliquoted (2 ml) into glass tubes, and biofilms were allowed to develop at 28°C, with 40 rpm shaking. After 4 h, the supernatants were taken out and the tubes were rinsed three times with distilled water. Then 2.5 ml 0.1% CV was added into each tube to stain for 10 min. The excess stain was washed off three times with distilled water. The CV that stained the biofilm was destained with 3 ml of 95% ethanol by leaving it at room temperature for 20 min. Then optical density (OD_590_) of the destained solution was examined, using a spectrophotometer (INESA). The absorbance value was positively correlated to the amount of biofilm. All tests were performed in triplicate and the absorbance readings were averaged.

### 2.5 Assays for β‐galactosidase activity

β–galactosidase activity was determined from sodium dodecyl sulfate‐ and chloroform‐permeabilized cells as described (Miller, 1992). Overnight cultures were inoculated (1:100 dilution) in fresh LB medium and grown for 1.5 h; cultures were diluted 1:1 three times (every half hour) before the start of sample collection. These steps were done to ensure proper dilution of β‐galactosidase accumulated after overnight growth. Experiments were repeated at least three times with two technical repeats per sample, and data are given in Miller units.

### 2.6 RNA extraction and primer extension

Total RNA from exponentially growing cells in LB medium was extracted with total RNA extraction reagent (Vazyme) as recommended by the manufacturer. Following extraction, total RNA was treated with DNaseI, which was later heat‐inactivated at 70°C for 15 min. The transcriptional start site was determined using the 5′ rapid amplification of cDNA ends (5′ RACE) system, as recommended by the supplier (Clontech, www.bdbiosciences.com) with 3 μg of total RNA (DNA‐free) obtained above. The gene‐specific primer *bifA‐*race (Table 1) was used to initiate the first‐strand cDNA synthesis. Small aliquots of cDNA as templates were amplified using SMART PCR primer mix, UP, and gene‐specific primer *bifA‐*race (Table 1). The resulting PCR product was cloned into the T‐easy vector (Takara) according to the manufacturer's instructions before being sequenced.

### 2.7 RNA extraction, preparation of cDNA and qRT‐PCR

Total RNA from exponentially growing cells in LB medium was extracted with total RNA extraction reagent (Vazyme) as recommended by the manufacturer. Reverse transcription reactions to generate the corresponding cDNA were performed with 1 μg of RNA using PrimeScriptTM RT reagent kit (Takara RR047A). RpoD was used as an internal control for normalization (Savli et al., 2003). Sequence of primer used for qRT‐PCR (qpcrbifAs, qpcrbifAa) was listed in Table 1. The results were analyzed by means of the comparative threshold cycle method to determine the relative expression of *bifA* gene in target strains with respect to the wild type with empty vector. Three individual replicates were performed with three independent cultures grown on different days. Standard errors were calculated from these independent replicates.

### 2.8 Extraction and quantification of intracellular c‐di‐GMP

C‐di‐GMP was extracted and quantitated as previously described (Morgan, Kohn, Hwang, Hassett, & Sauer, 2006; Petrova, Schurr, Schurr, & Sauer, 2011; Roy, Petrova, & Sauer, 2013). Briefly, c‐di‐GMP was extracted in triplicate from wild‐type and mutant strains grown planktonically to exponential phase using heat and ethanol precipitation followed by centrifugation. Supernatants were combined, dried using a Speed‐Vac and resuspended in deionized water. Samples (30 μl) were analyzed using an Agilent 1260 HPLC equipped with an autosampler, degasser, and Targa column (2.1 × 40 mm; 5 μm) at a detector set to 253 nm, and separated using a reverse‐phase C_18_ flow rate of 0.2 ml/min with the following gradient: 0 to 6.3 min, 2% B; 6.3 to 11 min, 10% B; 11 to 13.5 min, 90% B; 13.5 to 18 min, 2% B (buffer A, 10 mM ammonium acetate; buffer B, methanol plus 10 mM ammonium acetate). Commercially available c‐di‐GMP was used as a reference for the identification and quantification of c‐di‐GMP in cell extracts. c‐di‐GMP levels were normalized to total protein per ml of culture.

### 2.9 Statistical analysis

All statistical analyses were performed in Microsoft Excel, using a two‐tailed Student's *t* test, assuming equal variance, or single‐factor analysis of variance (ANOVA). All experiments were performed at least three times. A *p* value less than or equal to.05 was considered as statistically significant.

## 3 Results and Discussion

### 3.1 Transcription of *bifA* decreased in *fliA* deletion mutant

**Figure 1:**
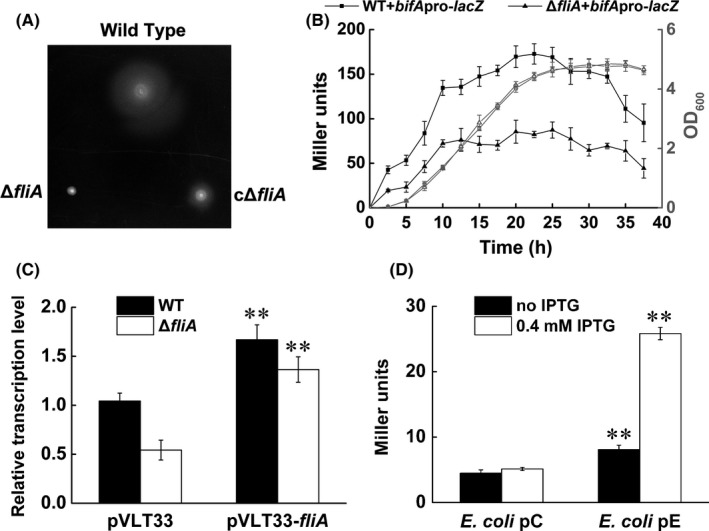
Influence of FliA deletion or overexpression on transcription of *bifA*. (A) Swimming assay. Wild‐type KT2440, *fliA* mutant and *fliA* mutant complement cells were spotted onto swimming plates contained 0.4 mM IPTG. The photograph was taken 20 hr after inoculation at 28°C. (B) Growth (open symbols) and β‐galactosidase activity (filled symbols) of wild type (squares) and the *fliA* deletion mutant (triangles) harboring the *bifA::lacZ* fusion in pBBR‐*bifA*pro‐*lacZ*. Results are averages and standard deviations from three experiments with duplicate samples. (C) Analysis of the influence of FliA overexpression on the transcription level of *bifA* by qRT‐PCR. Total RNA was extracted 3 hr after induction with 0.4 mM IPTG. Results are averages and standard deviations from three experiments with duplicate samples. **Statistically significant difference between FliA overexpression strain and the control strain (*p* < .01). (D) Heterologous expression of FliA in reporter *Escherichia coli* strain. Results are averages and standard deviations from three experiments with duplicate samples. **Statistically significant difference between β‐Galactosidase activities of pE and pC (*p *<* *.01)

To test whether transcription of *bifA* was controlled by FliA, we used allelic replacement to construct an unmarked deletion of *fliA*, and then promoter activity of *bifA* was compared between wild type and the *fliA* deletion mutant using promoter‐*lacZ* fusion plasmid. The mutant was nonmotile, as expected, and the motility defect was complemented with wild‐type copy of FliA expressed by the IPTG‐inducible *lacI*/P *tac* promoter of pVLT33 (Fig. 1A). Result of β‐galactosidase activity showed that deletion of *fliA* in *P. putida*, while not impacting growth rates in liquid LB broth (Fig. 1B), resulted in an about twofold decrease in activity of *bifA* promoter relative to the wild type. QRT‐PCR data revealed that complementation with wild‐type *fliA* restored the transcription level of *bifA* in the *fliA* mutant to a higher level than that observed in wild‐type strain with empty vector pVLT33 (Fig. 1C). Besides, overexpression of *fliA* induces the expression of *bifA* in both wild‐type and *fliA* deletion mutant strains (Fig. 1C).

FliA was one of 24 alternative σ‐factors of *P. putida* KT2440 (Martínez‐Bueno, Tobes, Rey, & Ramos, 2002) that conferred promoter‐recognition specificity to core RNA polymerase (RNAP) (Koo et al., 2009; Kuznedelov et al., 2002). In order to further verify the conclusion that FliA‐RNAP control transcription of *bifA*, an *E. coli* reporter strain was constructed by introducing the promoter‐*lacZ* fusion plasmid pBBR*‐bifApro‐lacZ* to *E. coli* DH5α. Then FliA expression vector pVLT33‐*fliA* and empty vector pVLT33 were introduced into the *E. coli* reporter strain, respectively, to generate *E. coli* pE and *E. coli* pC (control) strains. Although the promoter activity in *E. coli* was rather low relative to that in *P. putida*, after 3 hr induction with 0.4 mM IPTG, the β‐galactosidase activity of the strain *E. coli* pE was approximately fivefolds higher than that of the strain *E. coli* pC (Fig. 1D). Taken together, these results suggest that FliA control transcription of *bifA*.

Consistent with previously published results showing that transcription of *bifA* in a *fliA* mutant decreased approximately twofold in a microarray analysis (Rodríguez‐Herva et al., 2010), we observed twofold lower levels of *bifA* promoter transcription activity in *fliA* mutant compared with that of the wild type (Fig. 1B). Similar to the function of FliA in *E. coli*, FliA from *P. putida* also controlled expression of *yhjH*, coding a PDE involved in c‐di‐GMP turnover. In addition to *yhjH*, FliA controls expression of the c‐di‐GMP receptor YcgR in *E. coli K‐12* and *Chlamydia trachomatis* (Claret et al., 2007; Shen et al., 2006; Yu et al., 2006).

### 3.2 Identify the transcription start point of *bifA*

**Figure 2:**
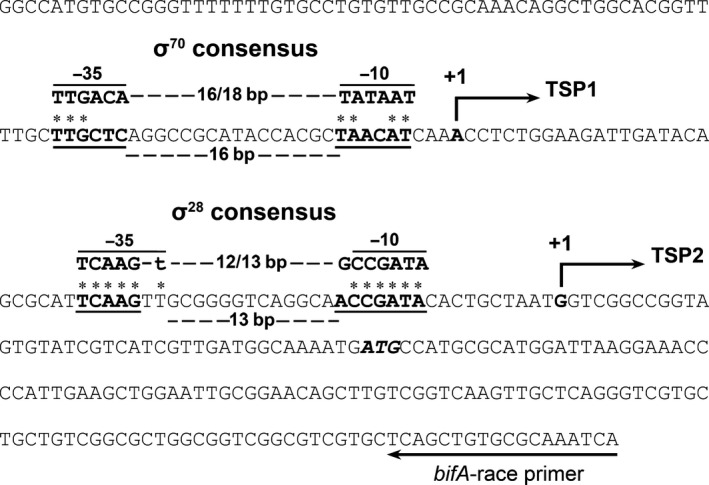
Identification of the *bifA* transcription start points (TSPs). Nucleotide sequence of the 150 bp region upstream of the *bifA* ATG initiation codon (bold italic) was shown. The putative σ^70^ and σ^28^ promoter sequences were indicated. The two TSPs were highlighted in bold and indicated by arrows

Promoter‐*lacZ* fusion assay revealed that FliA deletion led to a twofold decrease rather than a totally abolish to the *bifA* promoter activity, indicating that besides FliA other sigma factors may also be involved in *bifA* transcription. By analyzing the promoter sequence of *bifA*, we found a putative σ^70^ promoter sequence ahead of the putative σ^28^ promoter sequence (Mclean, Wiseman, & Kropinski, 1997; Rodríguez‐Herva et al., 2010) (Fig. 2), thus we hypothesized that transcription of *bifA* was controlled by two sigma factors that start from two or more transcription start points (TSPs). To test our hypothesis, 5′‐race was utilized to identify the TSP of *bifA*. As expected, two TSPs were found in the promoter region, located on 103 and 40 nt upstream of the translation initiation codon, and with the putative σ^70^ and σ^28^ promoter sequences upstream, respectively (Fig. 2). Location of the two TSPs indicates that transcription of *bifA* is controlled by σ^28^ and σ^70^, but whether σ^70^ participates in controlling *bifA* transcription or not still requires further verification.

Several examples of promoter sites that can be recognized by two or more sigma factors had been reported (Cao & Helmann, 2002; Jervis, Thackray, Houston, Horsburgh, & Moir, 2007; Minnig, Barblan, Kehl, Möller, & Mauël, 2003). The overlap between sigma factors may function as a regulation mode to modulate gene expression. We infer that σ^70^ acts as a housekeeping sigma factor to give a basic *bifA* expression level, while σ^28^ functions as an enhancement factor to increase transcription of *bifA* under certain conditions or at certain periods.

A recent study in *P. aeruginosa* PAO1 revealed that the FliA gene modulates the c‐di‐GMP concentration via *bifA* to regulate motility and phenazine pigment production (Lo et al., 2016), and suggested that this regulation may be indirect due to the lack of a canonical FliA promoter sequence in the promoter region of *bifA*. We also tried to compare the upstream regions of *bifA* in *P. putida* KT2440 with those in *P. aeruginosa* PAO1, but no significant similarity was found between the two sequences, and no σ^28^ consensus sequence was found in the *bifA* promoter region of the PAO1 strain (sequences of the two promoter were shown in Data S1). We infer that while function of FliA in controlling *bifA* may be conserved in the two *Pseudomonas* species, the σ^28^ promoter consensus sequences may be different. Future studies on determining the FliA promoter consensus sequence in PAO1 would help to answer this question.

### 3.3 Mutations in the putative σ^28^ promoter sequence decreased the *bifA* promoter activity

**Figure 3:**
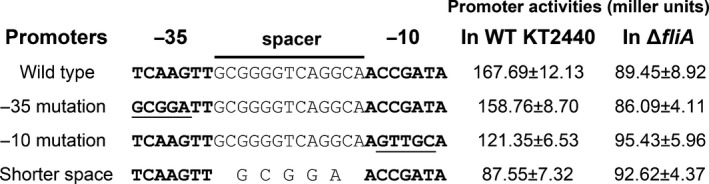
Influence of point mutations in the putative σ^28^ promoter sequence on the *bifA* promoter activity. β‐Galactosidase activities of wild type or mutated *bifA* promoter fusions, as indicated on the left of the diagram, in wild‐type KT2440 and *fliA* deletion mutant. ‐35 and ‐10 regions of the putative promoters were indicated with bold letters. Mutated sequences were indicated with underline. The β‐galactosidase values are indicated on the right and expressed as means ± standard deviation

To further characterize the capacity of FliA in regulating *bifA*, we created point mutations in the putative σ^28^ promoter sequence of the *bifA* promoter region and analyzed the influences of these mutations on promoter activity. As shown in Fig. 3, mutations located in ‐35, ‐10, and spacer (shorted from 13 to 5 bp) regions of the putative σ^28^ promoter were constructed, respectively, and then activities of these promoters were analyzed by the promoter‐*lacZ* fusion reporter plasmid and compared with that of the wild‐type promoter. Result of β‐galactosidase activity showed that the mutation in ‐35 region had no obvious impact on the promoter activity, the mutated promoter showed a transcriptional activity close to that of the wild‐type promoter in both wild‐type strain and the *fliA* deletion strain. However, both mutations in ‐10 and spacer regions significantly reduced the promoter activity in wild‐type strain background, indicating that the two mutations impaired the function of the promoter in the wild‐type strain background. Besides, both mutations in ‐10 and spacer regions showed no obvious influence on the promoter activity in the *fliA* deletion mutant, the two mutated promoters showed similar activities as that of the wild‐type promoter in the *fliA* deletion strain background. Compared with the mutations in ‐35 and ‐10 regions, mutation in the spacer region reduced about half of the transcriptional activity of the promoter in wild‐type KT2440, a level close to that caused by *fliA* deletion, indicating that the σ^28^ promoter was totally abolished by the mutation. These results further proved the function of the σ^28^ promoter and FliA controlled transcription of *bifA*.

### 3.4 FliA overexpression decreased intracellular c‐di‐GMP level via BifA

**Figure 4:**
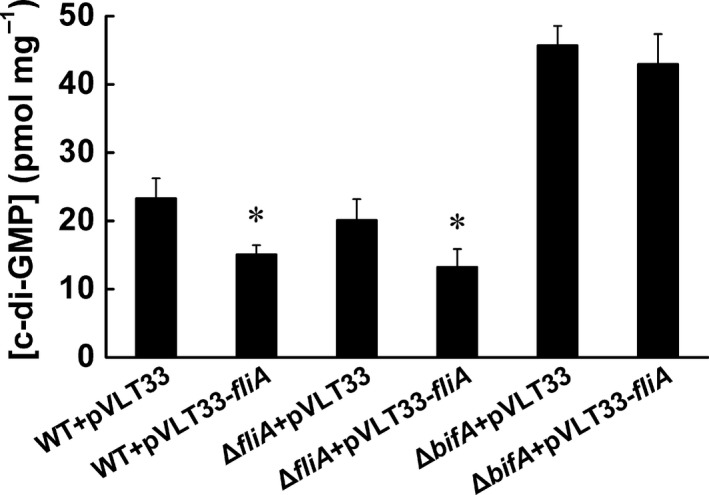
Quantification of intracellular c‐di‐GMP. c‐di‐GMP was extracted and measured as described in Materials and Methods. Strains are wild‐type KT2440, *fliA* mutant and *bifA* mutant, harboring pVLT33 or pVLT33‐*fliA*, respectively. Data represent averages of three independent cultures. *Significantly different between FliA overexpression strain (harboring pVLT33‐*fliA*) and the control strain (harboring pVLT33) (*p* < .05)

BifA was a PDE involved in the regulation of the intracellular c‐di‐GMP pool in KT2440 (Jiménez‐Fernández et al., 2015). Since the expression of *bifA* was partly under the control of FliA, loss or overexpression of FliA may influence the intracellular c‐di‐GMP level. To confirm our presumption, C‐di‐GMP levels of the wild‐type, *fliA* deletion mutant, and FliA overexpressing strains were measured using HPLC. Unexpectedly, no obvious difference in c‐di‐GMP concentrations was observed between wild‐type (23.33 pmol/mg protein) and the *fliA* mutant (20.13 pmol/mg protein) (Fig. 4). But in both strains, FliA overexpression caused an about 35% decrease in the c‐di‐GMP level. To determine whether modulation of c‐di‐GMP by FliA was BifA dependent, an unmarked *bifA* deletion mutant was constructed, and FliA overexpression vector or empty vector was introduced into the *bifA* mutant. Thereafter c‐di‐GMP levels were quantified. c‐di‐GMP concentration of *bifA* mutant was nearly twice as that in the wild type, about 45.73 pmol/mg protein. With the same amount of IPTG added to the culture, no obvious difference in c‐di‐GMP levels was observed between the FliA overexpression *bifA* mutant strain (42.98 pmol/mg protein) and the control *bifA* mutant strain (45.73 pmol/mg protein). This result indicated that *bifA* deletion abolished the influence of FliA on the c‐di‐GMP level.

Although expression of *bifA* was under the control of FliA, FliA deletion showed no obvious influence on the c‐di‐GMP level, the reason may lie in the functional redundancy of DGCs and PDEs in the strain. The genome of *P. putida* KT2440 encodes 43 polypeptides potentially involved in the c‐di‐GMP turnover (Ulrich & Zhulin, 2007). Relationship, expression, and coordination of these proteins must be a complex and extensive system. The effect of decreased *bifA* expression on the c‐di‐GMP level in the *fliA* mutant may be compensated by the function of other DGCs or PDEs.

Activity of FliA was inhibited by FlgM until the assembly of the hook‐basal body (HBB) was completed through which the FlgM was secreted (Chadsey et al., 1998). We speculated that the accumulation of FliA before HBB formation would cause a FliA overexpression effect after secretion of FlgM. Then free FliA drives the transcription of class IV flagellar genes to form complete flagella with other components. Meanwhile, free FliA enhances expression of BifA, and lowered the intracellular c‐di‐GMP level.

### 3.5 FliA overexpression enhanced swimming ability in wild‐type strain, but not in *bifA* mutant strain

**Figure 5:**
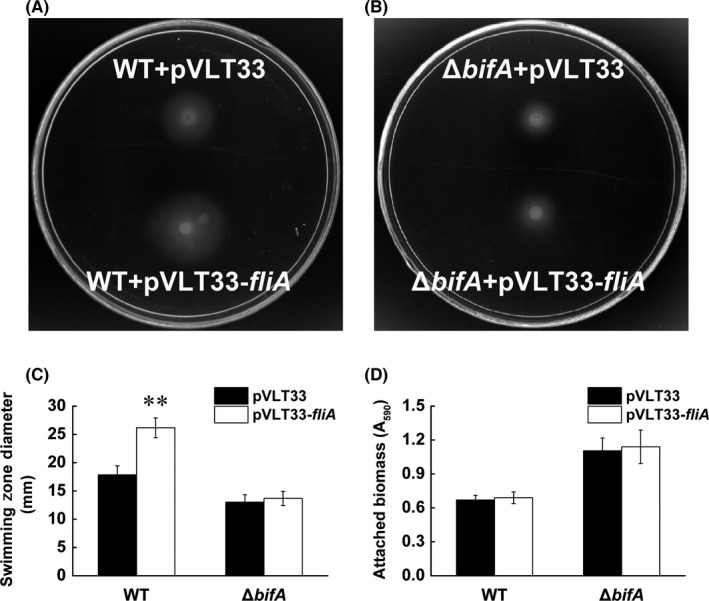
Influence of FliA overexpression on swimming ability and biofilm formation of wild‐type and *bifA* mutant. Wild‐type KT2440 (A) and *bifA* mutant (B), harboring pVLT33 or pVLT33‐*fliA* respectively, were spotted onto swimming plates contained 0.4 mM IPTG. The photograph was taken 20 hr after inoculation at 28°C. (C) Quantitative measurements of swimming zone areas are presented for four replicates of the assay shown in paned A and B. (D) Biofilm formation on glass tube surface was quantified by staining attached cells with the crystal violet method. The results are the average of three independent assays. **Statistically significant difference between FliA overexpression strain and the control strain (*p* < .05)

It has been reported that reducing the intracellular c‐di‐GMP level promotes swimming ability and inhibits biofilm formation of *P. putida* (Martínez‐Granero et al., 2014; Xiao et al., 2016). Since FliA overexpression decreased the c‐di‐GMP level in the wild type via BifA, we assumed that FliA overexpression could enhance swimming ability and inhibit biofilm formation in wild‐type strain, but not in the *bifA* mutant strain. To test our hypothesis, swimming ability and biofilm formation of wild type and *bifA* mutant both harboring pVLT33‐*fliA* was tested, strains harboring empty plasmid pVLT33 were used as control. As expected, swimming ability of wild type was enhanced by FliA overexpression (swimming zone measurements: WT + pVLT33 = 17.83 ± 1.61 mm, WT + pVLT33‐*fliA *= 26.17 ± 1.76 mm) (Fig. 5A and C), while that of the *bifA* mutant was not affected (swimming zone measurements: Δ*bifA *+ pVLT33 = 13 ± 1.32 mm, Δ*bifA* + pVLT33‐*fliA *= 13.67 ± 1.26 mm) (Fig. 5B and C). The *bifA* mutant strain with an empty vector showed weaker swimming motility than that of the wild type, probably due to the higher c‐di‐GMP level in the mutant. Biofilm formation assay showed that FliA overexpression did not influence biofilm formation in both the wild‐type strain and the *bifA* mutant, as the strains harboring FliA overexpression vector or control vector showed similar biofilm formation ability (Fig. 5D).

In general, increasing the cellular c‐di‐GMP levels induces the production of extracellular matrix components and increases biofilm formation, whereas low levels promote swimming motility. But little is known about the dosage effect of c‐di‐GMP in promoting motility or biofilm formation. Swimming motility is pushed by flagellar rotation, while biofilm formation is done by secretion of several matrix components and attaching to surfaces. In contrast to swimming ability, we confer that the effect of c‐di‐GMP reduction caused by FliA overexpression on biofilm formation is too weak to detect.

In line with a recent study in *P. aeruginosa* PAO1 revealed that FliA modulates the C‐di‐GMP concentration via *bifA* to regulate motility (Lo et al., 2016), we also found that FliA acted as a negative regulator to modulate the c‐di‐GMP level via controlling the transcription of *bifA* to facilitate motility in *P. putida* KT2440. Together with the former finding in *E. coli* (Claret et al., 2007), it seems that modulation of the c‐di‐GMP level by controlling expression of PDE coding genes is a conservative function of FliA and its homologs.

**Figure 6:**
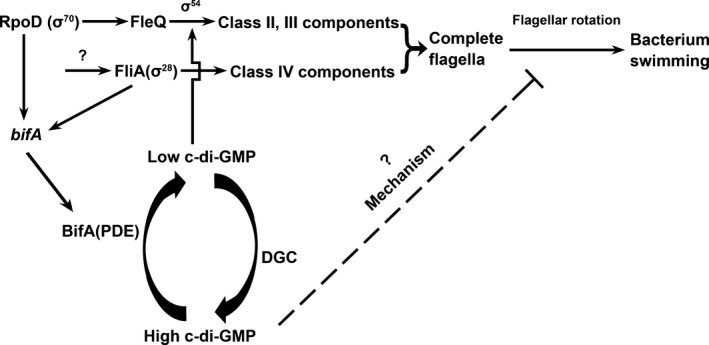
Model for FliA modulates intracellular c‐di‐GMP level via regulating BifA expression in *Pseudomonas putida* KT2440. After secretion of FlgM through hook‐basal body, free FliA leads to expression of class IV genes, and forms a complete flagellum together with other flagella components. Besides, FliA also caused a concomitant induction of the *bifA*. BifA degrades c‐di‐GMP to lower its concentration, which would favor expression of flagella genes via FleQ, and relieve the inhibition of flagella rotation caused by a high C‐di‐GMP level. The induction of BifA by FliA acts as a positive feedback loop to ensure a favorable condition for swimming by decreasing the intracellular c‐di‐GMP level

In summary, we showthat the expression of PDE BifA was partly controlled by the flagellar sigma factor FliA and FliA overexpression decreases the intracellular c‐di‐GMP level in *P. putida* KT2440. By enhancing the expression of BifA, FliA acted as a negative regulator to modulate the c‐di‐GMP level to facilitate swimming motility (Fig. 6). Our results indicated a positive feedback function of FliA in the swimming ability regulation.

## Funding Information

The research was financially supported by the National Natural Science Foundation of China (41571230, 41230854, J1103510), the National Basic Research Program of China (973, grant No. 2015CB150504), and the Fundamental Research Funds for the Central Universities (2662015PY016, 2662015PY116).

## Conflict of Interest

The authors declare that they have no competing interests.

## References

[mbo3402-bib-0001] Aldridge, P. & Hughes, K. T. (2002). Regulation of flagellar assembly. Current Opinion in Microbiology, 5, 160–165. 11934612 10.1016/s1369-5274(02)00302-8

[mbo3402-bib-0002] Bagdasarian, M. , Lurz, R. , Rückert, B. , Franklin, F. C. H. , Bagdasarian, M. M. , Frey, J. & Timmis, K. N. (1981). Specific‐purpose plasmid cloning vectors II. Broad host range, high copy number, RSF 1010‐derived vectors, and a host‐vector system for gene cloning in *Pseudomonas* . Gene, 16, 237–247. 6282695 10.1016/0378-1119(81)90080-9

[mbo3402-bib-0003] Baraquet, C. & Harwood, C. S. (2015). A FleQ DNA binding consensus sequence revealed by studies of FleQ‐dependent regulation of biofilm gene expression in *Pseudomonas aeruginosa* . Journal of Bacteriology, 198, 178–186. 26483521 10.1128/JB.00539-15 PMC4686206

[mbo3402-bib-0004] Cao, M. & Helmann, J. D. (2002). Regulation of the *Bacillus subtilis bcrC* bacitracin resistance gene by two extracytoplasmic function sigma factors. Journal of Bacteriology, 184, 6123–6129. 12399481 10.1128/JB.184.22.6123-6129.2002 PMC151963

[mbo3402-bib-0005] Chadsey, M. S. , Karlinsey, J. E. & Hughes, K. T. (1998). The flagellar anti‐sigma factor FlgM actively dissociates *Salmonella typhimurium* sigma28 RNA polymerase holoenzyme. Genes & Development, 12, 3123–3136. 9765212 10.1101/gad.12.19.3123 PMC317189

[mbo3402-bib-0006] Claret, L. , Miquel, S. , Vieille, N. , Ryjenkov, D. A. , Gomelsky, M. & Darfeuille‐Michaud, A. (2007). The flagellar sigma factor FliA regulates adhesion and invasion of Crohn disease‐associated *Escherichia coli* via a cyclic dimeric GMP‐dependent pathway. Journal of Biological Chemistry, 282, 33275–33283. 17827157 10.1074/jbc.M702800200

[mbo3402-bib-0007] Dasgupta, N. , Wolfgang, M. C. , Goodman, A. L. , Arora, S. K. , Jyot, J. , Lory, S. & Ramphal, R. (2003). A four‐tiered transcriptional regulatory circuit controls flagellar biogenesis in *Pseudomonas aeruginosa* . Molecular Microbiology, 50, 809–824. 14617143 10.1046/j.1365-2958.2003.03740.x

[mbo3402-bib-0008] De Lorenzo, V. , Eltis, L. , Kessler, B. & Timmis, K. N. (1993). Analysis of *Pseudomonas* gene products using *lacI* ^*q*^ */Ptrp‐lac* plasmids and transposons that confer conditional phenotypes. Gene, 123, 17–24. 8380783 10.1016/0378-1119(93)90533-9

[mbo3402-bib-0009] Fang, X. & Gomelsky, M. (2010). A post‐translational, c‐di‐GMP‐dependent mechanism regulating flagellar motility. Molecular Microbiology, 76, 1295–1305. 20444091 10.1111/j.1365-2958.2010.07179.x

[mbo3402-bib-0010] Frisk, A. , Jyot, J. , Arora, S. K. & Ramphal, R. (2002). Identification and functional characterization of *flgM*, a gene encoding the anti‐sigma 28 factor in *Pseudomonas aeruginosa* . Journal of Bacteriology, 184, 1514–1521. 11872701 10.1128/JB.184.6.1514-1521.2002 PMC134903

[mbo3402-bib-0011] Hengge, R. (2009). Principles of c‐di‐GMP signalling in bacteria. Nature Reviews Microbiology, 7, 263–273. 19287449 10.1038/nrmicro2109

[mbo3402-bib-0012] Hickman, J. W. & Harwood, C. S. (2008). Identification of FleQ from *Pseudomonas aeruginosa* as a c‐di‐GMP‐responsive transcription factor. Molecular Microbiology, 69, 376–389. 18485075 10.1111/j.1365-2958.2008.06281.x PMC2612001

[mbo3402-bib-0013] Jenal, U. & Malone, J. (2006). Mechanisms of cyclic‐di‐GMP signaling in bacteria. Annual Review of Genetics, 40, 385–407. 10.1146/annurev.genet.40.110405.090423 16895465

[mbo3402-bib-0014] Jervis, A. J. , Thackray, P. D. , Houston, C. W. , Horsburgh, M. J. & Moir, A. (2007). SigM‐responsive genes of *Bacillus subtilis* and their promoters. Journal of Bacteriology, 189, 4534–4538. 17434969 10.1128/JB.00130-07 PMC1913368

[mbo3402-bib-0015] Jiménez‐Fernández, A. , López‐Sánchez, A. , Calero, P. & Govantes, F. (2015). The c‐di‐GMP phosphodiesterase BifA regulates biofilm development in *Pseudomonas putida* . Environmental Microbiology Reports, 7, 78–84. 25870874 10.1111/1758-2229.12153

[mbo3402-bib-0016] Koo, B. M. , Rhodius, V. A. , Campbell, E. A. & Gross, C. A. (2009). Mutational analysis of *Escherichia coli* sigma28 and its target promoters reveal recognition of a composite ‐10 region, comprised of an “extended ‐10” motif and a core ‐10 element. Molecular Microbiology, 72, 830–843. 19400790 10.1111/j.1365-2958.2009.06691.x PMC2756079

[mbo3402-bib-0017] Kuchma, S. L. , Brothers, K. M. , Merritt, J. H. , Liberati, N. T. , Ausubel, F. M. & O'Toole, G. A. (2007). BifA, a cyclic‐Di‐GMP phosphodiesterase, inversely regulates biofilm formation and swarming motility by *Pseudomonas aeruginosa* PA14. Journal of Bacteriology, 189, 8165–8178. 17586641 10.1128/JB.00586-07 PMC2168662

[mbo3402-bib-0018] Kuznedelov, K. , Minakhin, L. , Niedziela‐Majka, A. , Dove, S. L. , Rogulja, D. , Nickels, B. E. , … Severinov, K. (2002). A role for interaction of the RNA polymerase flap domain with the sigma subunit in promoter recognition. Science, 295, 855–857. 11823642 10.1126/science.1066303

[mbo3402-bib-0019] Liu, R. & Ochman, H. (2007). Stepwise formation of the bacterial flagellar system. Proceedings of the National Academy of Sciences USA, 104, 7116–7121. 10.1073/pnas.0700266104 PMC1852327 17438286

[mbo3402-bib-0020] Lo, Y. L. , Shen, L. , Chang, C. H. , Bhuwan, M. , Chiu, C. H. & Chang, H. Y. (2016). Regulation of motility and phenazine pigment production by FliA is cyclic‐di‐GMP dependent in *Pseudomonas aeruginosa* PAO1. PLoS ONE, 11, e0155397. 27175902 10.1371/journal.pone.0155397 PMC4866697

[mbo3402-bib-0021] Martínez‐Bueno, M. A. , Tobes, R. , Rey, M. & Ramos, J. L. (2002). Detection of multiple extracytoplasmic function (ECF) sigma factors in the genome of *Pseudomonas putida* KT2440 and their counterparts in *Pseudomonas aeruginosa* PA01. Environmental Microbiology, 4, 842–855. 12534467 10.1046/j.1462-2920.2002.00371.x

[mbo3402-bib-0022] Martínez‐García, E. , Nikel, P. I. , Chavarría, M. & de Lorenzo, V. (2014). The metabolic cost of flagellar motion in *Pseudomonas putida* KT2440. Environmental Microbiology, 16, 291–303. 24148021 10.1111/1462-2920.12309

[mbo3402-bib-0023] Martínez‐Gil, M. , Ramos‐González, M. I. & Espinosa‐Urgel, M. (2014). Roles of cyclic Di‐GMP and the Gac system in transcriptional control of the genes coding for the *Pseudomonas putida* adhesins LapA and LapF. Journal of Bacteriology, 196, 1484–1495. 24488315 10.1128/JB.01287-13 PMC3993364

[mbo3402-bib-0024] Martínez‐Granero, F. , Navazo, A. , Barahona, E. , Redondo‐Nieto, M. , González de Heredia, E. & Baena, I. , et al. (2014). Identification of *flgZ* as a flagellar gene encoding a PilZ domain protein that regulates swimming motility and biofilm formation in *Pseudomonas* . PLoS ONE, 9, e87608. 24504373 10.1371/journal.pone.0087608 PMC3913639

[mbo3402-bib-0025] Mclean, B. W. , Wiseman, S. L. & Kropinski, A. M. (1997). Functional analysis of σ70 consensus promoters in *Pseudomonas aeruginosa* and *Escherichia coli* . Canadian Journal of Microbiology, 43, 981–985. 9396150 10.1139/m97-141

[mbo3402-bib-0026] Miller, J. H. (1992). A short course in bacterial genetics: a laboratory manual and handbook for Escherichia coli and related bacteria. New York: Cold Spring Harbor Laboratory Press.

[mbo3402-bib-0027] Minnig, K. , Barblan, J. L. , Kehl, S. , Möller, S. B. & Mauël, C. (2003). In *Bacillus subtilis* W23, the duet σ X σ M, two sigma factors of the extracytoplasmic function subfamily, are required for septum and wall synthesis under batch culture conditions. Molecular Microbiology, 49, 1435–1447. 12940998 10.1046/j.1365-2958.2003.03652.x

[mbo3402-bib-0028] Morgan, R. , Kohn, S. , Hwang, S. H. , Hassett, D. J. & Sauer, K. (2006). BdlA, a chemotaxis regulator essential for biofilm dispersion in *Pseudomonas aeruginosa* . Journal of Bacteriology, 188, 7335–7343. 17050921 10.1128/JB.00599-06 PMC1636253

[mbo3402-bib-0029] O'Toole, G. A. & Kolter, R. (1998). Initiation of biofilm formation in *Pseudomonas fluorescens* WCS365 proceeds via multiple, convergent signalling pathways: a genetic analysis. Molecular Microbiology, 28, 449–461. 9632250 10.1046/j.1365-2958.1998.00797.x

[mbo3402-bib-0030] Paul, K. , Nieto, V. , Carlquist, W. C. , Blair, D. F. & Harshey, R. M. (2010). The c‐di‐GMP binding protein YcgR controls flagellar motor direction and speed to affect chemotaxis by a “backstop brake” mechanism. Molecular Cell, 38, 128–139. 20346719 10.1016/j.molcel.2010.03.001 PMC2929022

[mbo3402-bib-0031] Petrova, O. E. , Schurr, J. R. , Schurr, M. J. & Sauer, K. (2011). The novel *Pseudomonas aeruginosa* two‐component regulator BfmR controls bacteriophage mediated lysis and DNA release during biofilm development through PhdA. Molecular Microbiology, 81, 767–783. 21696457 10.1111/j.1365-2958.2011.07733.x PMC3214647

[mbo3402-bib-0032] Rodríguez‐Herva, J. J. , Duque, E. , Molina‐Henares, M. A. , Navarro‐Avilés, G. , Van Dillewijn, P. & De La Torre, J. , et al. (2010). Physiological and transcriptomic characterization of a *fliA* mutant of *Pseudomonas putida* KT2440. Environmental Microbiology Reports, 2, 373–380. 23766109 10.1111/j.1758-2229.2009.00084.x

[mbo3402-bib-0033] Roy, A. B. , Petrova, O. E. & Sauer, K. (2013). Extraction and quantification of cyclic di‐GMP from *Pseudomonas aeruginosa* . Bio‐protocol, 3, e828. 25429368 10.21769/bioprotoc.828 PMC4241849

[mbo3402-bib-0034] Savli, H. , Karadenizli, A. , Kolayli, F. , Gundes, S. , Ozbek, U. & Vahaboglu, H. (2003). Expression stability of six housekeeping genes: a proposal for resistance gene quantification studies of Pseudomonas aeruginosa by real‐time quantitative RT‐PCR. Journal of Medical Microbiology, 52, 403–408. 12721316 10.1099/jmm.0.05132-0

[mbo3402-bib-0035] Shen, L. , Feng, X. , Yuan, Y. , Luo, X. , Hatch, T. P. & Hughes, K. T. , et al. (2006). Selective promoter recognition by chlamydial sigma28 holoenzyme. Journal of Bacteriology, 188, 7364–7377. 16936033 10.1128/JB.01014-06 PMC1636291

[mbo3402-bib-0036] Simon, R. , Priefer, U. & Pühler, A. (1983). A broad host range mobilization system for in vivo genetic engineering: transposon mutagenesis in gram negative bacteria. Nature Biotechnology, 1, 784–791.

[mbo3402-bib-0037] Ulrich, L. E. & Zhulin, I. B. (2007). MiST: a microbial signal transduction database. Nucleic Acids Research, 35, D386–D390. 17135192 10.1093/nar/gkl932 PMC1747179

[mbo3402-bib-0038] Xiao, Y. , Nie, H. , Liu, H. , Luo, X. , Chen, W. & Huang, Q. (2016). C‐di‐GMP regulates the expression of *lapA* and *bcs* operons via FleQ in *Pseudomonas putida* KT2440. Environmental Microbiology Reports, doi:10.1111/1758‐2229.12419. 10.1111/1758-2229.12419 27120564

[mbo3402-bib-0039] Yu, H. H. Y. , Kibler, D. & Tan, M. (2006). In silico prediction and functional validation of σ^28^‐regulated genes in *Chlamydia* and *Escherichia coli* . Journal of Bacteriology, 188, 8206–8212. 16997971 10.1128/JB.01082-06 PMC1698183

