# Supplementary material for: Expression of the phosphodiesterase BifA facilitating swimming motility is partly controlled by FliA in Pseudomonas putida KT2440
Source: Microbiologyopen. 2016 Sep 23;6(1):e00402. doi: 10.1002/mbo3.402 (PMC5300878; doi:10.1002/mbo3.402)
Supplement: Supplementary file 1 [file MBO3-6-0-s001.docx]

**Supplementary Information**

**Sequences of *bifA* promoter in *P. putida* KT2440 and *P. aeruginosa* PAO1**

*BifA* promoter in KT2440

AGTCATTCGGCCTTGAACGAGAAACCCGGCCATGTGCCGGGTTTTTTTGTGCCTGTGTTGCCGCAAACAGGCTGGCACGGTTTTGC**TTGCTC**AGGCCGCATACCACGC**TAACAT**CAAACCTCTGGAAGATTGATACAGCGCAT**TCAAGTT**GCGGGGTCAGGCA**ACCGATA**CACTGCTAATGGTCGGCCGGTAGTGTATCGTCATCGTTGATGGCAAAATG***ATG***CCATGC

*BifA* promoter in PAO1

TGGTCAACTGGGACTTCGTAGCGAAGAATTTCGCTGCCTGAGTCTGATCAGAACCACGAAAAAGCCCGGCATTCGCCGGGCTTTTTCGTTTTAATCTGCGGTCGAGGAGCCATCGTCTACCCTCAGGAACTGGTAGACCTTCGCTCGTGATAGCCTCTTGAGGTAAACAAGCTCTTCCTACCGGCTTGTCTTTCGCCCTGGCGACGAACAAGGAAGGCCCC***TTG***AAACTG

Initiation codons (ATG and TTG) were shown in bold italic. The putative σ^70^ and σ^28^ promoter sequences in KT2440 were indicated with bold. Sequences were retrieved from the *Pseudomonas* genome database website (http://www.pseudomonas.com/).
